# The interaction between sarcoplasmic reticulum and mitochondria: a novel mechanism for cardiac arrhythmia

**DOI:** 10.3389/fphys.2026.1777143

**Published:** 2026-03-16

**Authors:** Zhongyang Yu, Meng Zhao, Hongyue Xu, Ji Sun, Xiaoxing Jin

**Affiliations:** 1 State Key Laboratory of Cardiovascular Diseases, Shanghai East Hospital, School of Medicine, Tongji University, Shanghai, China; 2 Shanghai Frontiers Center of Nanocatalytic Medicine, Shanghai, China; 3 Department of Pathology and Pathophysiology, School of Medicine, Tongji University, Shanghai, China; 4 Clinical Center for Heart Disease Research, Tongji University, Shanghai, China; 5 Shanghai Arrhythmia Research Center, Shanghai East Hospital, School of Medicine, Tongji University, Shanghai, China; 6 Department of Cardiology, Shanghai East Hospital, School of Medicine, Tongji University, Shanghai, China; 7 State Key Laboratory for Diagnosis and Treatment of Severe Zoonotic Infectious Diseases, Key Laboratory for Zoonosis Research, Ministry of Education, Institute of Zoonosis, College of Veterinary Medicine, Jilin University, Changchun, China; 8 Department of Cardiology, the First Affiliated Hospital, Sun Yat-sen University, Guangzhou, Guangdong, China; 9 Department of Cardiovascular Medicine, Fifth Affiliated Hospital of Sun Yat-sen University, Zhu Hai, China; 10 Department of Cardiology of Central China Fuwai Hospital, Central China Fuwai Hospital of Zhengzhou University, Zhengzhou, China

**Keywords:** a novel mechanism, cardiac arrhythmia, mitochondria, sarcoplasmic reticulum, SR-mitochondria interaction

## Abstract

Cardiovascular diseases are a major cause of morbidity and mortality worldwide. Cardiac arrhythmias, especially fatal ventricular arrhythmias, are highly harmful to patients and can even lead to sudden death. While electrical and structural remodeling of myocardial tissue represent the mainstream mechanisms underlying arrhythmogenesis, there is a critical need to explore novel perspectives. This review focuses on the communication between the sarcoplasmic reticulum and mitochondria as an independent mechanistic lens. We detail how this specific interaction governs Ca^2+^ transfer and cell-death signaling, positioning it as a potentially pivotal, distinct pathway that contributes to and amplifies the development of cardiac arrhythmias.

## 1 Introduction

Cardiac arrhythmias are a group of diseases characterized by heart rhythm disorders. Severe arrhythmias usually lead to cardiac dysfunction and even sudden death (Zeppenfeld et al., 2022; Bariani et al., 2024; Wolfe et al., 2024). Consequently, the severity of these conditions has garnered significant attention from clinical experts worldwide. However, our understanding of the mechanisms underlying the onset and development of arrhythmias remains limited. Previous studies have established two main pathological mechanisms underlying cardiac arrhythmias: electrical remodeling and structural remodeling (Sagris et al., 2021; Wijesurendra and Casadei, 2019). Recently, growing research into interactions between organelles, such as the endoplasmic reticulum (ER) and mitochondria, have provided a new perspective for interpreting the pathological mechanisms of cardiac arrhythmias (Boyman et al., 2020; Wiersma et al., 2019). In cardiomyocytes, the ER is referred to as the sarcoplasmic reticulum (SR), which is responsible for Ca^2+^ storage, release, and uptake. SR–mitochondria interactions modulate multiple physiological processes in cardiomyocytes, including Ca^2+^ transfer (Adamson et al., 2024; Agyapong et al., 2024; Bertero et al., 2021), energy metabolism, and cell death (Clements et al., 2023; Goodman et al., 2020; Maiuolo et al., 2021). Furthermore, the alterations of SR-mitochondria interactions contributed to cardiac remodeling, which finally lead to cardiovascular diseases (Nichtová et al., 2023; Gomez et al., 2016). In this review, we summarize how the SR and mitochondria interact and how their perturbation contributes to and exacerbates the development of arrhythmias. Moreover, we discuss potential molecules involved in SR–mitochondria interactions as therapeutic targets for arrhythmias.

## 2 The mechanisms for cardiac arrhythmia

Cardiac remodeling is generally considered a response to physiological or pathological stimulation (Schüttler et al., 2019; Nakamura and Sadoshima, 2018). Initially, it acts as a compensatory process to maintain cardiac function. However, long-term remodeling results in decompensation and, ultimately, heart failure (Frantz et al., 2022); for instance, prolonged and intensive training may also be associated with an elevated risk of cardiovascular diseases (Luan et al., 2021). Therefore, clinicians often regard arrhythmia-associated remodeling as a crucial pathological process, and various therapies have been developed to reverse its detrimental effects.

### 2.1 Electrical remodeling

As a muscular organ, the heart is mainly comprised of cardiomyocytes and fibroblasts in myocardial tissue. In healthy cardiomyocytes, when the plasma membrane depolarizes and L-type calcium channels (LTCCs) open, Ca^2+^ flows into the cytoplasm and induces Ca^2+^ release mediated by ryanodine receptor 2 (RyR2) from the SR (Eisner et al., 2017). Then, the dramatic increase in Ca^2+^ concentration triggers sarcomere contraction, and finally the heart beats rhythmically. This process is also called excitation–contraction coupling in cardiomyocytes. Meanwhile, electrical signals conduct from one cardiomyocyte to another through intercellular gap junctions, and cardiomyocytes are electrically activated in sequence. However, electrical activation and propagation in the myocardium are remodeled and disordered in arrhythmia. The mechanism behind the electrical activation and propagation is the alterations in density, kinetics and function of key types of channels. These changes are defined as electrical remodeling.

The sinoatrial node (SAN) is the origin of cardiac rhythm in the whole heart. Pacemaker cells in the SAN regulate heart rate through spontaneous depolarization. This phenomenon mainly relies on the funny current (I_f_) generated by the hyperpolarisation-activated cyclic nucleotide-gated channel 4 (HCN4) channel protein in the plasma membrane. In addition to aging, underlying diseases like diabetes are common causes of sinus arrhythmia, as they can trigger electrical remodeling and structural remodeling in the SAN (Hennis et al., 2024). Moreover, long-term high-glucose environment induces SR stress, leading to the accumulation of unfolded or misfolded proteins within the SR, thereby promoting cardiomyocyte apoptosis, activating cardiac fibroblasts, exacerbating cardiac fibrosis and leading to arrhythmia (Mantovani et al., 2022). Hyperlipidemia is the primary cause of atherosclerosis. Severe atherosclerosis can also induce myocardial oxidative stress and cardiomyocyte death, leading to structural remodeling characterized by cardiac fibrosis and ultimately inducing various types of arrhythmias (Li et al., 2025). In diabetes, I_f_ is upregulated, attributed to increased fatty acids and sympathetic tone, and induces Ca^2+^ overload in pacemaker cells (Vinogradova et al., 2002; Deng et al., 2012). A clinical study indicated that the expression level of another cardiac sodium channel, Nav1.5, also determined heart rate in patients with sinus arrhythmia (Ferdous et al., 2016). Certain pathways have been shown to participate in sinoatrial node electrophysiology. Zheng et al. reported that Hippo–Yap signaling was necessary for Ca^2+^ homeostasis in pacemaker cells and maintained sinus node function (Zheng et al., 2022). Except for Hippo–Yap, overactivation of Notch signaling changed the expression of voltage-gated sodium channel alpha subunit 5 (Scn5a) and gap junction protein alpha 5 (Gja5) at the transcriptional level in sick sinus syndrome (Qiao et al., 2017). In summary, alterations in the expression levels of ion channels such as HCN4 and Nav1.5, as well as dysregulation of sodium, calcium, and gap junction protein expression mediated by the Hippo-Yap and Notch signaling pathways, are the primary causes of sinus arrhythmia.

In addition to the sinoatrial node, structural remodeling also occurs in other regions of the heart, such as the atria and ventricles, which finally contribute to arrhythmia. Atrial fibrillation (AF) is the most common cardiac arrhythmia. In patients with AF, ectopic impulses are generated from the atrium rather than the sinus node and spread to other areas in the heart. Nearly 95% of ectopic impulses originate from the pulmonary veins (Haïssaguerre et al., 1998). As a result, ablation of the pulmonary veins has been a primary treatment for AF. Pulmonary vein ablation has become the cornerstone of catheter ablation for treating AF and is widely recommended as a first-line therapy for symptomatic paroxysmal AF patients; however, adverse effects still exist, including pericardial tamponade, stroke or transient ischemic attack, and esophageal injury/atrio-esophageal fistula (Rottner and Metzner, 2023). Numerous studies have investigated the molecular targets of AF in the past decades to explain how ectopic impulses originate. Since the first familial AF gene, KCNQ1, was identified in 2003 (Chen et al., 2003), many sodium channels (Olesen et al., 2012; Li et al., 2013), potassium channels (Ni et al., 2017; Christophersen et al., 2013), and calcium channels (Weeke et al., 2014; McCauley et al., 2020) have been confirmed to be involved in the onset of AF. Among these channels, calcium channels are widely studied because Ca^2+^ handling disorders serve as an important cause of AF. Studies have shown that ectopic activity in the atrium could be related to early afterdepolarizations (EADs) and delayed afterdepolarizations (DADs). Under pathological conditions, in atrial cardiomyocytes, elevated diastolic Ca^2+^ leak from the SR mediated by RyR2 dominantly contributes to these afterdepolarizations, and then action potential duration (APD) is shortened, which finally leads to AF (Nattel et al., 2020). Clinical studies revealed that RyR2 is a crucial pro-arrhythmic gene in patients with diverse types of AF (Voigt et al., 2014; Voigt et al., 2012; Heijman et al., 2020). Increased expression and activity of RyR2 could promote AF by enhancing Ca^2+^ leak (Beavers et al., 2013; Chiang et al., 2014). Pitx2 is a gene encoding a homeodomain-containing transcription factor (Bai et al., 2021). Pitx2 mutations affect Cav1.2 (L-type calcium channel), while also lead to abnormal sarcoplasmic reticulum (SR) calcium handling (Fang et al., 2022). In addition, homozygous knockout of Pitx2 at the gene level can elevate AF susceptibility by increasing Ca^2+^ signaling and pharmacological inhibition of RyR2 remarkably suppressed AF occurrence after Pitx2 deficiency (Kim et al., 2023). Besides RyR2, SERCA2a is another core gene modulating Ca^2+^ homeostasis. During diastole, SERCA2a re-uptakes nearly 70% of Ca^2+^ in the cytoplasm back into the SR. In preclinical mouse models, SERCA2a upregulation by transgenic techniques in mice model improved Ca^2+^ release in RyR2-associated AF (Liu et al., 2020). Likewise, reduced SERCA2a activity has been found to promote the occurrence of postoperative AF by inducing Ca^2+^ overload. Details about how RyR2 and SERCA2a regulate Ca^2+^ homeostasis and how the activities of these two channels are modulated will be discussed in later sections (Liu et al., 2020; Fakuade et al., 2021). Overall, electrical remodeling, especially Ca^2+^ mishandling in atrial cardiomyocytes, is the primary pathological mechanism for AF.

Similar to AF, electrical property alterations during ventricular arrhythmia can also be attributed to disordered Ca^2+^ handling. During ventricular arrhythmias such as ventricular fibrillation (VF), store-overload-induced Ca^2+^ release (SOICR) could trigger Ca^2+^ waves, increase the excitability of cardiomyocytes, and finally cause malignant ventricular tachycardia (Priori and Chen, 2011). Early in this century, a series of clinical studies explored the potential mechanisms for polymorphic ventricular tachycardia, and researchers identified RyR2 mutations, such as R176W, E2296K, and V2193L, as a molecular characteristic in patients with polymorphic ventricular tachycardia (Laitinen et al., 2001; Priori et al., 2002; Lahat et al., 2003; Lehnart et al., 2004a). Patients carrying these mutations tended to show more spontaneous Ca^2+^ release events and higher susceptibility to VF compared to healthy people (Paavola et al., 2007). In animal experiments, research from Chen et al. showed that loss-of-function mutations in RyR2 at amino acid E4872 occur at the exact site for Ca^2+^ activation and completely blocked SOICR events (Chen et al., 2014). Given that the E4872 residue of the RyR2 gene is highly conserved during evolution (Uchinoumi et al., 2025), diagnostic genetic testing targeting RyR2 mutations—including the E4872 site—has been incorporated into the molecular genetic screening guidelines for CPVT patients (Failla et al., 2024). Wang et al. (2020) found that RyR2 opening was significantly facilitated by deficiency of integrin β1D in a model of arrhythmogenic right ventricular cardiomyopathy. Since Ca^2+^ disorders are common in ventricular arrhythmia, there will be more evidence about the role of RyR2 in the disease. In conclusion, electrical remodeling such as Ca^2+^ mishandling is an indispensable reason for cardiac arrhythmia.

### 2.2 Structural remodeling

**FIGURE 1:**
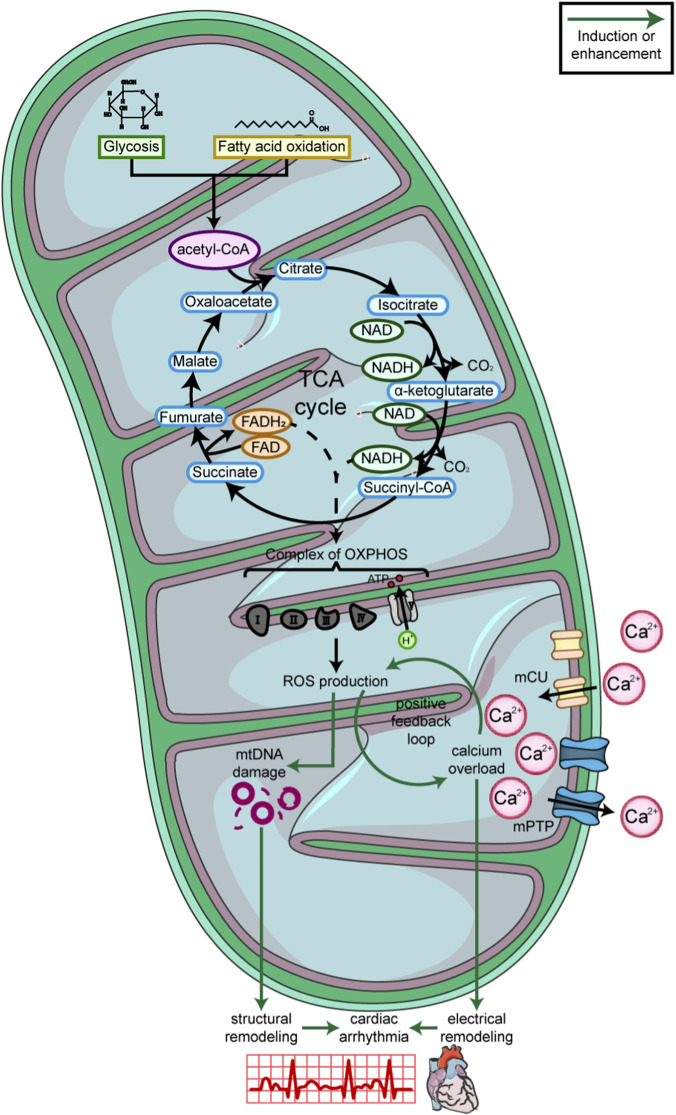
Representative diagram about the mechanisms of structural remodeling in cardiac arrhythmia. Fibrosis can be divided into two types in cardiac arrhythmia: reparative and interstitial fibrosis. Multiple cell death types of cardiomyocytes trigger reparative fibrosis, while interstitial fibrosis is a response to stress. Under mechanical stress like stretch, or facing neurohumoral stimulation factors like TGF-β, angiotensin II (AngII), endothelin 1 (ET-1) and IL-6, fibroblasts will be activated and induce structural remodeling in cardiac arrhythmia.

Structural remodeling is another aspect during the development of cardiac arrhythmia (Figure 1). Electrical remodeling is mostly more prominent and common in the early stages of arrhythmia, while the role of structural remodeling gradually increases and becomes a key factor in maintaining the arrhythmia (Bai et al., 2021). Therefore, cardiac structural remodeling are more severe in elders because of aging related diseases like hypertension, diabetes, coronary heart disease and heart failure (Gibb and Hill, 2018). Evidence from patients with cardiac arrhythmia, especially older individuals, indicates that structural remodeling characterized by fibrosis is common in heart rhythm disorders (Akoum et al., 2018; Laredo et al., 2018). A recent clinical study combined transmission electron microscopy (TEM), immunohistochemistry, and electroanatomic mapping to explore histopathological and electrophysiological changes in patients undergoing AF ablation and endomyocardial atrial biopsy, and the researchers confirmed that atrial fibrosis was increased, intercellular spaces were enlarged, and myocardial fibers were disrupted in these patients’ hearts (Takahashi et al., 2023). The impact of myocardial fibrosis on arrhythmias is extensive and profound. First, myocardial fibrosis causes electrical conduction abnormalities, impeding the normal propagation of electrical signals and resulting in slowed conduction velocity or conduction block (Verheule and Schotten, 2021). The electromechanical interaction between cardiomyocytes and fibroblasts can also affect myocardial function (Kursanov et al., 2023). Furthermore, myocardial fibrosis leads to the formation of a re-entrant substrate in the myocardial tissue, creating areas of slow conduction and functional conduction block. This provides an ideal substrate for the initiation and maintenance of re-entrant arrhythmias, such as atrial fibrillation and ventricular tachycardia. Particularly in the border zones of scars after myocardial infarction, the interweaving of fibrotic tissue and surviving myocardium is highly prone to forming re-entrant circuits (Masè et al., 2024). Simultaneously, myocardial fibrosis significantly slows conduction velocity and may shorten the effective refractory period, thereby shortening the wavelength of the electrical impulse. This allows smaller anatomical regions to sustain re-entrant circuits.

Currently, the main clinical methods for detecting myocardial fibrosis are endocardial voltage mapping and imaging assessments, while histological methods such as Masson’s trichrome staining are used in animal experimental models (Ravassa et al., 2023). According to endocardial voltage mapping and imaging assessments in arrhythmia patients, when the fibrosis density reaches above 30%–40% of the local myocardial area, the conduction velocity can be reduced by more than 50% (Quah et al., 2021).

Although there are currently no approved targeted anti-fibrotic drugs that can directly reverse established fibrosis, several strategies and drugs show potential in delaying fibrosis progression. These include adhering to the treatment of underlying diseases, RAAS inhibitors, SGLT2 inhibitors, and drugs targeting key fibrotic signaling pathways such as TGF-β (Psotka et al., 2019).

In fact, fibrosis can be divided into two types: reparative and interstitial fibrosis (Frangogiannis, 2019). With respect to reparative fibrosis, when the heart is injured acutely, such as that occurring after myocardial infarction, cardiomyocytes are damaged or die, and fibrosis is required to fill the space in order to maintain organ integrity (Nattel, 2017). Several types of cell death, including apoptosis (Weng et al., 2023), necroptosis (Kang et al., 2020), pyroptosis (Yuan et al., 2023) and ferroptosis (Su et al., 2023) have been observed and validated in cardiomyocytes. All these types of cell death are followed by significant pathological fibrosis. Although reparative fibrosis is a “filling” process aimed at maintaining organ integrity following cardiomyocyte injury or death, excessive reparative fibrosis leads to scar formation. These scar tissues not only lack contractile function but may also create abnormal electrical conduction pathways, thereby establishing the pathological substrate for the initiation and maintenance of arrhythmias (Stratton and McKinsey, 2016). Hence, molecules regulating cell fate such as TGF-β and Wnt/β-catenin also participate in the process of cardiac structural remodeling, and they are vital to the occurrence of cardiac arrhythmia (Damani and Topol, 2009). With respect to interstitial fibrosis, it has been regarded as a response to chronic stress (Brundel et al., 2022), which has been well demonstrated in arrhythmias accompanied by metabolic diseases. Cardiac fibrosis is significantly increased in diabetes (Li et al., 2023), whereas antidiabetic drugs, for example, dapagliflozin, are effective in preventing these alterations (Lee et al., 2022; Tian et al., 2021).

As about 50%–60% of cells in the heart are fibroblasts, they are the most abundant cells in cardiac tissue, and their activation is a core mechanism of cardiac fibrosis (Nattel, 2018). Activated fibroblasts proliferate rapidly, synthesize a large amount of fibrous protein in the myocardium, and promote the deposition of extracellular matrix (Spencer et al., 2017). In the early stages of myocardial fibrosis, excessive deposition of collagen occurs (Neuber et al., 2021). In the middle stages of myocardial fibrosis, the activation of fibroblasts and structural remodeling lead to myocardial stiffness and diastolic dysfunction *via* fibroblast growth factor 23 (FGF-23) and related signaling pathways, marking the onset of arrythmia and heart failure (Camelliti and Stuckey, 2023). In the late stages of myocardial fibrosis, arrhythmia and full-blown heart failure develops, potentially leading to sudden cardiac death (McKinsey et al., 2023). Although it is a crucial process for structural support and damage repair in the heart, excessive fibroblast activation decreases conduction velocity and facilitates substrate formation under conditions of cardiac arrhythmia such as AF (Spronk et al., 2017).

Fibroblasts can be stimulated by mechanical stress, such as pressure or stretch (Davis and Molkentin, 2014; Verheule et al., 2004). Meanwhile, fibroblasts are sensitive to multiple neurohumoral factors from nearby cells such as cardiomyocytes, as well as from distant organs under stress. TGF-β is a typical stimulator that influences gene expression in fibroblasts and aggravates cardiac fibrosis *via* Smad-associated pathways (Gould et al., 2012; Meng et al., 2023; Su et al., 2020). The signaling pathway constituted by hyaluronic acid (HA), CD44, and their downstream effectors—commonly referred to as the HA/CD44/STAT3 axis—represents a critical molecular framework in cancer biology. Moreover, TGF-β administration activates fibroblasts through the HA/CD44/STAT3 pathway and increases AF susceptibility (Chang et al., 2017). Electrical stimulation in neonatal rat ventricular cardiomyocytes induced the secretion of TGF-β and promoted the expression of collagen I and collagen III in neonatal fibroblasts (Slawik et al., 2019). In addition, loss of Hippo in cardiomyocytes also promoted fibroblast activation *via* TGF-β, disturbing the propagation of electrical activation from the sinoatrial node to the atrial myocardium (Liu H. et al., 2023). Furthermore, angiotensin II (AngII) and endothelin-1 (ET-1) from the circulatory system exhibit pro-fibrotic functions during the development of cardiac arrhythmia, whereas calcitonin from cardiomyocytes is able to inhibit cardiac fibrosis and fibroblast proliferation (Tsai et al., 2008; Li et al., 2005; Moreira et al., 2020).

The immune system is also crucial for cardiac fibrosis in the myocardium. During cardiac repair, immune cells are mobilized and recruited to injured myocardial tissue (Chen et al., 2019; Beiert et al., 2018). Macrophages in the myocardium, transformed from circulating monocytes, are crucial in regulating the activity of cardiac fibroblasts by secreting diverse cytokines (Kang et al., 2023), thereby modulating substrate formation for the onset of arrhythmia. Macrophages can polarize into distinct subtypes, including M1 (pro-inflammatory) and M2 (anti-inflammatory/pro-repair), depending on their microenvironment and functional state (Koga et al., 2021; Zhuang et al., 2024). The cytokines secreted by these different macrophage subsets exert varying effects on cardiac fibrosis. M1-type macrophages, play a pivotal role in the initiation and progression of cardiac fibrosis by secreting pro-inflammatory cytokines (Chen et al., 2019). TGF-β secreted by M1-type macrophages promotes the differentiation of cardiac fibroblasts into myofibroblasts and upregulates the expression of collagen type I (COL1A1) and type III (COL3A1), thereby enhancing extracellular matrix deposition (Frangogiannis, 2022). In addition, IL-1β derived from M1 macrophages has been found to exacerbate cardiac fibrosis by activating the NF-κB signaling pathway (Guo et al., 2024). Moreover, IL-1β secreted by macrophages in epicardial adipose tissue is also associated with postoperative atrial fibrillation and atrial remodeling (Cabaro et al., 2022). Additionally, M1-type macrophages produce TNF-α (Yap et al., 2023) and CCL2 (C-C motif chemokine ligand 2) (Shen et al., 2024) after myocardial injury and promote fibrosis in myocardium. IL-10 secreted by M2-type macrophages is a crucial anti-inflammatory cytokine. It inhibits the TGF-β/Smad signaling pathway and downregulates the expression of α-smooth muscle actin (α-SMA) and collagen, thereby reducing fibrosis (Dyachkova et al., 2023). In a mouse model of hyper-glycaemia induced by streptozotocin, pacemaker cells were surrounded by infiltrated fibrous tissue, and conduction velocity was decreased. Oxidative stress, inflammation, and fibrosis in the sinus node were ameliorated after administration of the cytokine IL-10 (Kondo et al., 2019). This study proved the efficacy of anti-inflammatory therapies in treating structural remodeling. In conclusion, physicochemical stress-induced fibroblast activation is a main driver of structural remodeling in cardiac arrhythmia.

Although advanced and extensive fibrosis (such as that found in patients with end-stage heart failure) is generally difficult to fully reverse (Edavettal and Gardner, 2022), structural remodeling induced by early-stage, mild cardiac fibrosis may be partially reversible; consequently, numerous studies are exploring potential therapeutic targets and strategies for myocardial fibrosis. Currently, TGF-β signaling pathway inhibitors (such as oleanolic acid derivatives) represent one of the primary anti-fibrotic strategies. Secondly, Renin-Angiotensin-Aldosterone System inhibitors (e.g., ACEIs/ARBs) and aldosterone receptor antagonists have been proven to improve myocardial fibrosis. Additionally, drugs targeting inflammatory responses and oxidative stress constitute another important direction for anti-fibrotic therapy (Travers et al., 2022).

Electrical and structural remodeling in arrhythmias not only serve as the foundation for the initiation and maintenance of AF, but their ultimate consequences also lead to further cellular remodeling. These processes exhibit significant similarities to the mechanisms of various other cardiac injuries or cardiovascular events (Hu et al., 2015; Korantzopoulos et al., 2018). First, if arrhythmias caused by long-term structural and electrical remodeling occur, they may increase the workload on cardiomyocytes, leading to cardiomyocyte hypertrophy. Meanwhile, factors such as ischemia, inflammation, and oxidative stress can also induce cardiomyocyte apoptosis, reducing the number of cardiomyocytes and further exacerbating atrial dysfunction (Zhou and Dudley, 2020). Furthermore, chronic arrhythmias can cause mitochondrial dysfunction, reducing ATP production and increasing the generation of reactive oxygen species (ROS), thereby exacerbating oxidative stress (Scott et al., 2018; Wang et al., 2021). Structural remodeling, electrical remodeling, and the resulting cellular remodeling show striking similarities to the mechanisms involved in other cardiac injuries or cardiovascular events. In heart failure, myocardial infarction, hypertensive heart disease, and diabetic cardiomyopathy, varying degrees of cardiomyocyte hypertrophy, oxidative stress, and apoptosis caused by structural and electrical remodeling are also observed (Frangogiannis, 2021).

## 3 SR and cardiac arrhythmia

**FIGURE 2:**
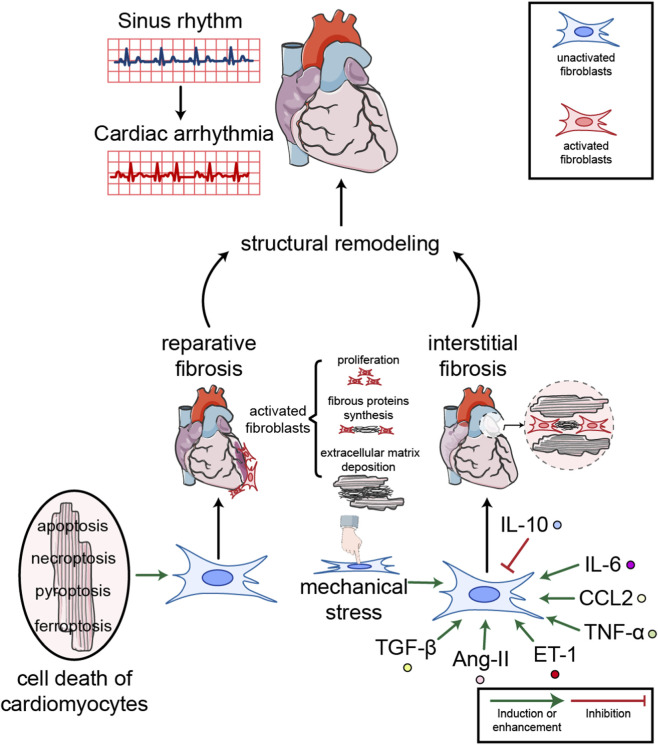
Representative diagram about the function of SR in atrial and ventricular arrhythmia. Calcium channels in SR are the gates for the Ca^2+^ releases and uptakes. In atrial cardiomyoctes, RyR2 can be activated when phosphorylated by Ca^2+^/calmodulin-dependent protein kinase II (CAMKII) with or without NLRP3 associated inflammation and PKA, while it can be stablized by interaction with calstabin2, phosphorylated by SPEG and de-phosphorylated protein phosphatase 1 (PP-1). Moreover, the phosphorylation of SERCA2 by JNK2 in atrium increased its activity (above). In ventricular cardiomyoctes, phosphorylation of RyR2 can also be increased by PKA or CaMKII related pathway. In contrast, RyR2 can be dephosphorylated by protein phosphatase 2A (PP2A). In the meanwhile, peptide AIP targeting CAMKII can inhibit the activity of RyR2 (below). Disorders in the modulation of RyR2 and SERCA2 will lead to Ca^2+^ mishandling and cardiac arrhythmia.

SR is an organelle involved in protein processing, modification, and quality control. In addition, lipid biogenesis and Ca^2+^ homeostasis are also associated with the SR (Figure 2). As mentioned above, SR Ca^2+^-handling proteins regulate Ca^2+^ release and uptake; thus, we summarized how these proteins are modulated under conditions of cardiac arrhythmia. For example, RyR2 can be activated when phosphorylated by Ca^2+^/calmodulin-dependent protein kinase II (CaMKII) (Uchinoumi et al., 2016; Curran et al., 2007) or PKA (Wei et al., 2023). NLRP3 is a key multiprotein complex within the innate immune system and it can also interact with CaMKII, which reflected the crosstalk between inflammation and electrical remodeling (Li et al., 2020). In atrial cardiomyocytes from patients with postoperative AF, the NLRP3/CaMKII nexus promoted spontaneous Ca^2+^ release and afterdepolarizations through RyR2 hyperphosphorylation (Cheng et al., 2022). Moreover, genetic inhibition of CaMKII and pharmacological inhibition of CaMKII by inhibitor KN-93 significantly prevented diastolic Ca^2+^ leak from the SR and decreased the AF induction rate in atrial cardiomyocytes from gene-edited mice and *ex vivo* human atrial cardiomyocytes (Neef et al., 2010; Li et al., 2012). It is important to note that while KN-93 has shown promise in preclinical and *ex vivo* studies, it has not been used therapeutically in human patients.

PKA is another kinase that phosphorylates RyR2. Calstabin2, also called FKBP12.6, is a natural stabilizer of RyR2 in cardiomyocytes from mammals (Wehrens et al., 2004; Gellen et al., 2008; Lehnart et al., 2004b). PKA levels were increased in the hearts of canines and patients with chronic AF, whereas PKA-phosphorylated RyR2 decreased the amount of calstabin2 interacting with RyR2, as examined by immunoprecipitation (Vest et al., 2005). In contrast to CaMKII and PKA, kinases such as SPEG exerted inhibitory effects on RyR2 *via* phosphorylation at S2367 (Campbell et al., 2020; Campbell et al., 2021). Genes encoding certain phosphatases can also modulate RyR2 phosphorylation levels and thereby influence AF occurrence. Loss of the protein phosphatase 1 (PP-1) subunit PPP1R3A increased the activities of RyR2 and SERCA2a and aggravated Ca^2+^ overload in atrial cardiomyocytes (Alsina et al., 2019). Phosphorylation of SERCA2 (a key SR Ca^2+^ pump) induced by JNK2 enhanced Ca^2+^ overload and promoted AF as well (Yan et al., 2021).

RyR2 phosphorylation plays a comparable role in ventricular cardiomyocytes to that in atrial cardiomyocytes. CaMKII has been confirmed to phosphorylate RyR2, and this modification promotes fatal ventricular arrhythmias (van Oort et al., 2010). Activated CaMKII, mediated by ROS, could phosphorylate RyR2 at S2814 in Barth syndrome induced by tafazzin mutation and lead to Ca^2+^ mishandling (Liu X. et al., 2021). Gene therapy targeting CaMKII, through AAV injection expressing a CaMKII inhibitory peptide (AIP) in cardiomyocytes, has been developed to treat catecholaminergic polymorphic ventricular tachycardia (CPVT) with RyR2 mutation (Bezzerides et al., 2019). More recently, Shi et al. reported that an AKT1/NOS3/CaMKII/RyR2 pathway was regulated by insulin receptor substrate protein-2 (IRS2) in ventricular cardiomyocytes, and IRS2 prevented catecholamine-sensitive ventricular tachycardia by governing Ca^2+^ homeostasis (Shi et al., 2024).

In contrast, RyR2 can be dephosphorylated by protein phosphatase 2A (PP2A) (Tsuji et al., 2011), and this regulation was reversed in mice with the ankyrin-B Q1283H mutation (Zhu et al., 2018). However, not all dephosphorylation events on RyR2 inhibit its activity. Endogenous PP-1 was reported to dephosphorylate RyR2 at S2808A and promote Ca^2+^ release in the ventricle (Potenza et al., 2020). Although SERCA2a is widely explored in cardiac electrophysiology, its function and regulation in ventricular arrhythmias are still not well understood. A study in 2004 indicated that constitutive SERCA2a overexpression exacerbated acute ventricular arrhythmias in rats with myocardial ischemia (Chen et al., 2004). However, SERCA2a overexpression prevented ventricular arrhythmias in a porcine model of ischemia–reperfusion and in guinea pigs with heart failure (Prunier et al., 2008; Cutler et al., 2012). Although there is a tendency suggesting that SERCA2a overexpression may be a potential therapy to protect the heart from sudden death, further investigation of SERCA2a function in models of ventricular arrhythmia is still urgently needed (Shah and Wasserstrom, 2012). In contrast to SERCA2a, therapeutic agents aimed at stabilizing the RyR2 channel (such as the 1,4-benzothiazepine derivative JTV519) have shown promise in animal models by preventing calstabin2 dissociation and normalizing calcium handling, thereby protecting the heart against arrhythmias (Wehrens et al., 2004). Currently, the perception regarding which approach holds more promise is nuanced. RyR2 inhibition offers a targeted approach to address the fundamental cause of Ca^2+^ leak, which directly triggers arrhythmias. In-depth understanding of RyR2 structure and its pathological modifications provides clear molecular targets for drug development (Miotto et al., 2024). However, the specificity and potential off-target effects of RyR2 inhibitors require careful consideration. In contrast, SERCA2a overexpression aims to restore overall calcium homeostasis, which can have broader benefits for cardiac function in preclinical studies. However, the inconsistent results regarding SERCA2a overexpression indicate that although it holds potential, a deeper investigation into its application within different arrhythmia contexts is still required, and its clinical application is not yet established. Overall, the SR functions as a key component modulating Ca^2+^ homeostasis in cardiomyocytes and thereby influences cardiac arrhythmias.

## 4 Mitochondria and cardiac arrhythmia

Mitochondria are the center of ATP synthesis in cardiomyocytes, and fatty acid oxidation is the main source of energy production (McLelland et al., 2023). Meanwhile, glycolysis also partially provides ATP in the heart. Through glycolysis, glucose is converted into pyruvate, which is then converted to acetyl-CoA; fatty acid oxidation produces acetyl-CoA as well. Next, acetyl-CoA enters the TCA cycle. As a result, nicotinamide adenine dinucleotide (NADH) and flavin adenine dinucleotide (FADH2) are generated (Nolfi-Donegan et al., 2020). Oxidative phosphorylation (OXPHOS) is the key biochemical process that produces ATP. The OXPHOS system comprises five multiprotein complexes (Complexes I–V). During OXPHOS, NADH and FADH2 donate electrons to the mitochondrial electron transport chain (ETC.), which are transferred through the respiratory complexes to ultimately reduce O_2_ (Zhao et al., 2019). Finally, ATP is produced by Complex V, utilizing the energy stored in the proton gradient across the inner mitochondrial membrane (i.e., in the intermembrane space) (Rahman et al., 2014). ATP is widely used for cellular activities such as sarcomere contraction and the operation of ion channels in cardiomyocytes.

Reactive oxygen species (ROS) are byproducts of OXPHOS due to electron leak from the, ETC and subsequent reactions with O_2_ (Schofield and Schafer, 2020). When mitochondrial dysfunction occurs because of aging, metabolic diseases, severe inflammatory diseases, and other causes, ROS production increases and impairs mitochondrial OXPHOS, which further promotes ROS production (Roohi et al., 2023).

The impact of mitochondria on cardiac electrophysiology can be divided into two parts. First, ROS can influence the activity of Ca^2+^ channels in the SR, which we will discuss later in this review. Second, mitochondria also function as an intracellular Ca^2+^ buffer and modulate Ca^2+^ homeostasis. As mentioned above, during diastole, Ca^2+^ is mostly reuptaken into the SR by SERCA. In addition, the Na^+^/Ca^2+^ exchanger (NCX) (Kohlhaas and Maack, 2010) and the mitochondrial Ca^2+^ uniporter (MCU) also play roles during this period. NCX in the plasma membrane extrudes Ca^2+^ from the cell, whereas MCU, together with its regulatory proteins (e.g., MICU1 and MICU2), takes up a portion of Ca^2+^ into mitochondria (Liu C. et al., 2023); mitochondrial Na^+^/Ca^2+^ exchange serves as a major route for mitochondrial Ca^2+^ efflux (Sisalli et al., 2014). Under physiological conditions, mitochondrial Ca^2+^ extrusion functions properly, and a defined amount of Ca^2+^ is taken up into mitochondria. However, in arrhythmias accompanied by elevated cytosolic Ca^2+^, NCX becomes impaired, resulting in increased mitochondrial Ca^2+^ accumulation and elevated ROS production. In turn, MCU can be activated by ROS, further promoting mitochondrial Ca^2+^ overload (Dong et al., 2017). Together, these studies demonstrate a positive feedback loop between mitochondrial Ca^2+^ and ROS. Given that NCX and MCU may influence arrhythmogenesis by affecting mitochondrial function and calcium overload, both have been regarded as therapeutic targets for arrhythmias. Currently, NCX as a potential antiarrhythmic target has been extensively studied, whereas MCU is considered an emerging target, with the development of its inhibitors still in the early stages. As a selective NCX blocker, SEA0400 has been proven to effectively inhibit NCX and influence the action potential. Another selective NCX blocker, ORM-10962, has also shown the ability to suppress repolarization alternans, suggesting its potential in preventing arrhythmias (Szlovák et al., 2020). Regarding MCU, its inhibitor Ru360 prevents the occurrence of ventricular arrhythmias by inhibiting mitochondrial Ca^2+^ transport, thereby preserving mitochondrial function and membrane integrity (Salazar-Ramírez et al., 2025). Although neither has been approved by the FDA or EMA as an official arrhythmia treatment target, both NCX and MCU demonstrate considerable promise.

**FIGURE 3:**
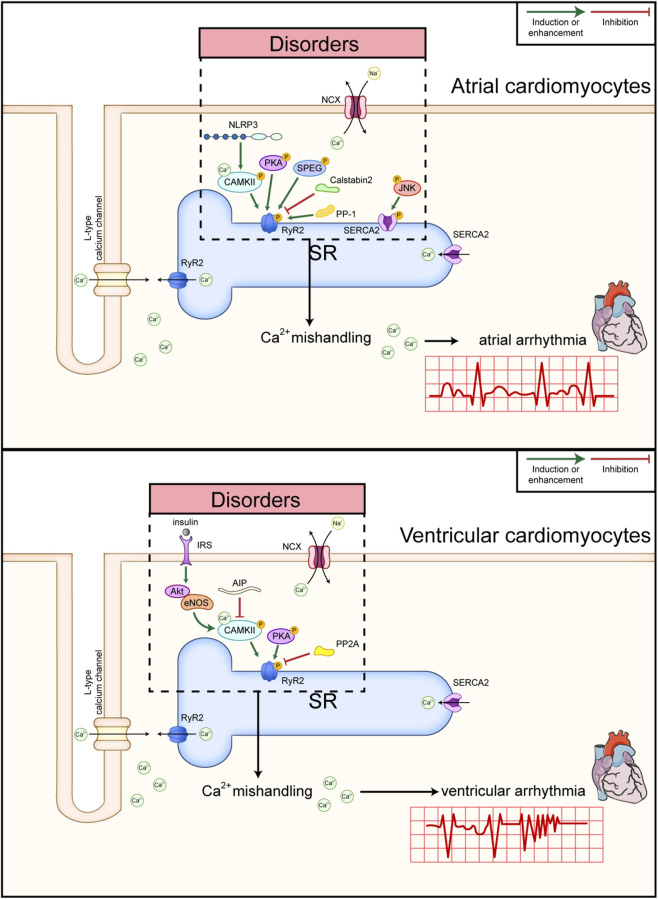
Representative diagram about the function of mitochondria in cardiac arrhythmia. Glycolysis and fatty acid oxidation provide acetyl-CoA for the TCA cycle, where nicotinamide adenine dinucleotide (NADH) and flavin adenine dinucleotide (FADH2) are synthesized. Oxidative phosphorylation (OXPHOS) consumes NADH and FADH2 with five multiprotein complexes including complex I/II/III/IV/V. Finally ATP is produced with byproducts named reactive oxygen species (ROS). Ca^2+^ concentration in mitochondria can influence ROS production and in return ROS production can modulate Ca^2+^ concentration in mitochondria *via* mitochondrial Ca^2+^ uniplex (mCU), which form a feedback loop and induce electrical remodeling in cardiomyocytes. ROS induced mitochondrial DNA (mtDNA) damage leads to structural remodeling as well. The electrical remodeling and structural remodeling contribute to cardiac arrhythmia at last.

Evidence from heart failure showed that arrhythmia susceptibility markers, including altered Ca^2+^ currents and increased EADs, were exacerbated by mitochondrial Ca^2+^ and ROS overload (Xie et al., 2018). Furthermore, mitochondrial ROS accumulates with age in older patients with AF, and the altered electrical properties of their atrial cardiomyocytes may be related to mitochondrial dysfunction (He et al., 2023). Interestingly, mitochondrial DNA (mtDNA) damage is another proposed mechanism for AF. A clinical prospective analysis based on multi-ethnic patients at risk of AF revealed that mtDNA copy number was negatively correlated with AF incidence. The authors predicted that mtDNA damage–induced mitophagy impaired the Ca^2+^-buffering function of mitochondria and ROS elimination (Zhao et al., 2020). In summary, as the factory of ATP production, mitochondria influence cardiac arrhythmia through a Ca^2+^–ROS feedback loop (Figure 3).

## 5 SR-mitochondria crosstalk in cardiac arrhythmias: a contributing mechanism at the Intersection of calcium signaling and redox regulation

In recent years, inter-organelle communication has attracted considerable attention, and SR–mitochondria interactions are among the most actively investigated areas, with increasing evidence highlighting their potential roles (Wang et al., 2022; Xiao et al., 2025). Multiple non-genetic causes induced by lifestyle and environmental factors can lead to alterations in sarcoplasmic reticulum (SR)-mitochondria interactions in cardiomyocytes, ultimately resulting in cardiovascular diseases (CVDs). The interface of SR-mitochondria interaction serves as a crucial site for non-vesicular lipid transport between the sarcoplasmic reticulum and mitochondria. Long-term high-fat diets can cause lipid metabolism disorders, potentially altering the lipid composition and flux at the SR-mitochondria contact sites. This leads to abnormal lipid accumulation, impairing mitochondrial function and cellular homeostasis, thereby promoting cardiomyocyte injury (Luan et al., 2021; Zhang et al., 2025). As an independent risk factor for CVDs, hyperglycemia may cause abnormalities of Ca^2+^ transport and lipid metabolism in SR-mitochondria contact sites, thus promoting cardiomyocyte injury (Ding et al., 2024). The SR-mitochondria contact site is also a regulatory center for the generation and scavenging of reactive oxygen species (ROS). Oxidative stress caused by poor lifestyle habits such as staying up late and chronic psychological stress may directly damage structural proteins at the SR-mitochondria contact sites, such as glucose-regulated protein 75 (GRP75) and mitofusin 2 (MFN2). This results in decreased stability of the connections between the SR and mitochondria, imbalanced Ca^2+^ transport, and consequently triggers mitochondrial dysfunction and cellular injury (Chen et al., 2024). Furthermore, long-term oxidative stress may lead to abnormal calcium transport mediated by the SR-mitochondria contact sites, causing mitochondrial calcium overload. This activates the mitochondrial permeability transition pore (mPTP), thereby promoting cardiomyocyte apoptosis. In summary, non-genetic factors such as sleep disorders and unhealthy diets directly or indirectly cause dysregulation of SR-mitochondrial interactions in cardiomyocytes through multiple mechanisms. Using fluorescence and electron microscopy, the molecular architecture at SR–mitochondria contact sites has been characterized. Mitochondria-associated endoplasmic reticulum membranes (MAMs), which physically link the SR and mitochondria, which enable rapid Ca^2+^ transfer from the SR *via* RyR2 or IP3 receptors to mitochondria *via* VDAC-MCU complexes. One well-defined structure is a complex formed by the SR Ca^2+^ channel inositol 1,4,5-trisphosphate receptor (IP3R) and the mitochondrial voltage-dependent anion channel (VDAC), bridged by the chaperone GRP75 (Szabadkai et al., 2006). Through this complex, Ca^2+^ can be transferred from the SR to mitochondria. Similarly, VDAC1 can form a complex with RyR2 to modulate mitochondrial Ca^2+^ entry (Kohlhaas and Maack, 2013), whereas IP3R can form a complex with FUNDC1 to mediate Ca^2+^ release (Wu et al., 2017). In addition, mitofusin 2 (MFN2) forms dimers spanning the SR and mitochondria and is involved in mitochondrial dynamics. Inverted formin 2 (INF2), which interacts with actin, is also a regulator of mitochondrial dynamics (Korobova et al., 2013). Different molecular machineries operating at SR-mitochondria contact sites function in a regulated manner rather than being constitutively active, thereby ensuring specialized and tightly controlled responses: The formation of MFN2 dimers spanning the SR and mitochondrial membranes is context-dependent and subject to regulation (Gottschalk et al., 2022). MFN2 plays a pivotal role in maintaining stable SR-mitochondria contacts, which undergo reorganization in response to alterations in cellular status (e.g., during SR stress). INF2-mediated SR-mitochondria tethering, which involves interaction with actin, is not constitutive but is triggered by specific stimuli such as Ca^2+^ spikes and RhoA signaling. This feature renders it a responsive mechanism for organelle remodeling (Hemel et al., 2021). The IP3R–FUNDC1 complex is specifically engaged during hypoxia, coupling Ca^2+^ signaling to mitophagy (Ray et al., 2023). Under hypoxic conditions, the interaction between IP3R and FUNDC1 facilitates the coordinated clearance of damaged mitochondria, indicating a stress-responsive function rather than continuous activity. The dysregulation of ER-mitochondria contact sites and their associated molecular machineries is linked to various diseases. This underscores that these systems are highly regulated and recruited during specific physiological or stress conditions, rather than functioning as continuously active units. The precise coordination of these complexes enables cells to maintain Ca^2+^ homeostasis, energy production, and overall cellular health (Katona et al., 2022). Together, these molecules maintain an SR–mitochondria distance of ∼33 nm and influence cellular functions including Ca^2+^ transfer, lipid exchange, signaling scaffolding, and organelle dynamics.

Physically, MCU can open when the local Ca^2+^ concentration rises to 20–30 μM (Marchi and Pinton, 2014). When RyR2 opens, the local Ca^2+^ concentration can increase to ∼30 μM, which is sufficient to activate MCU (Drago et al., 2012). β-adrenergic activation has been widely shown to increase SR Ca^2+^ release upon catecholamine stimulation in cardiomyocytes (Papa et al., 2022). Administration of catecholamines significantly elevated mitochondrial Ca^2+^ levels and respiration rates (Wu et al., 2015). Together, these findings indicate that mitochondrial metabolism is tightly regulated by SR Ca^2+^ release.

Conversely, mitochondria regulate SR function with respect to Ca^2+^ homeostasis. By finely controlling Ca^2+^ influx and efflux, mitochondria serve as an intracellular Ca^2+^ buffer (Maack and O'Rourke, 2007). An experiment in H9C2 cells suggested that mitochondria can sequester over 25% of intracellular Ca^2+^ (Pacher et al., 2000). In addition, MCU markedly influences Ca^2+^ transfer from the SR to mitochondria (Dorn and Maack, 2013).

Beyond their role as Ca^2+^ buffers, mitochondria are a major source of cellular ROS. In healthy cardiomyocytes, a ROS–Ca^2+^ feedback loop provides a mechanistic link between metabolism and cardiac rhythm. A 2022 study proposed that glutamate-induced ROS governed heart rate through oxidative activation of RyR2, SERCA2a, and CaMKII (Xie et al., 2022). Indeed, RyR2, SERCA2a, and CaMKII frequently undergo redox modifications in cardiovascular diseases such as myocardial ischemia, aging, and CPVT (Dries et al., 2018; Cooper et al., 2013; Liu H. et al., 2021). ROS can directly modulate RyR2 function because RyR2 contains abundant cysteine residues that are sensitive to oxidation (Nikolaienko et al., 2018), and ROS can also affect RyR2 activity indirectly. Genetic inhibition of mPTP promoted mitochondrial ROS production and induced RyR2 hyperphosphorylation, ultimately exacerbating arrhythmias (Deb et al., 2023). Hamilton et al. found that Ca^2+^ leak induced mitochondrial ROS production, and the increased ROS further activated RyR2, forming a positive feedback loop for Ca^2+^ release (Hamilton et al., 2020). Consistently, free-radical scavengers such as mercaptopropyl glycine reversed the pro-arrhythmic effects of ROS-mediated RyR2 modification (Becerra et al., 2016). Furthermore, dantrolene, primarily known for its direct action on RyR2 to prevent excessive Ca^2+^ release from the SR, modulates intracellular Ca^2+^ homeostasis, potentially protecting mitochondria from dysfunction and reducing apoptosis and myocardial fibrosis in conditions such as myocardial infarction (Xi et al., 2018). Ranolazine inhibits the late sodium current (late I_Na_), thereby reducing intracellular Na^+^ overload. This action indirectly impacts SR-mitochondria crosstalk by diminishing the reverse mode operation of the Na^+^/Ca^2+^ exchanger (NCX) and subsequent Ca^2+^ influx, ultimately alleviating cytosolic Ca^2+^ overload (Rouhana et al., 2021). Additionally, experimental compounds like MitoQ (Supinski et al., 2009) and SS-31 (Du et al., 2024) scavenge reactive oxygen species (ROS) in the vicinity of the mitochondrial inner membrane, leading to a reduction in myocardial fibrosis. In addition, increased oxidative stress can also prime the immune response to increase arrhythmia risk. For example, increased ROS production should prime and trigger the activation of the NLRP3 inflammasome, the components of which are upregulated in the atrial tissue of patients with POAF (Dobrev et al., 2019). In atrial fibrillation, anti-inflammatory strategies can reduce AF burden despite not correcting the primary Ca^2+^ defect.

**FIGURE 4:**
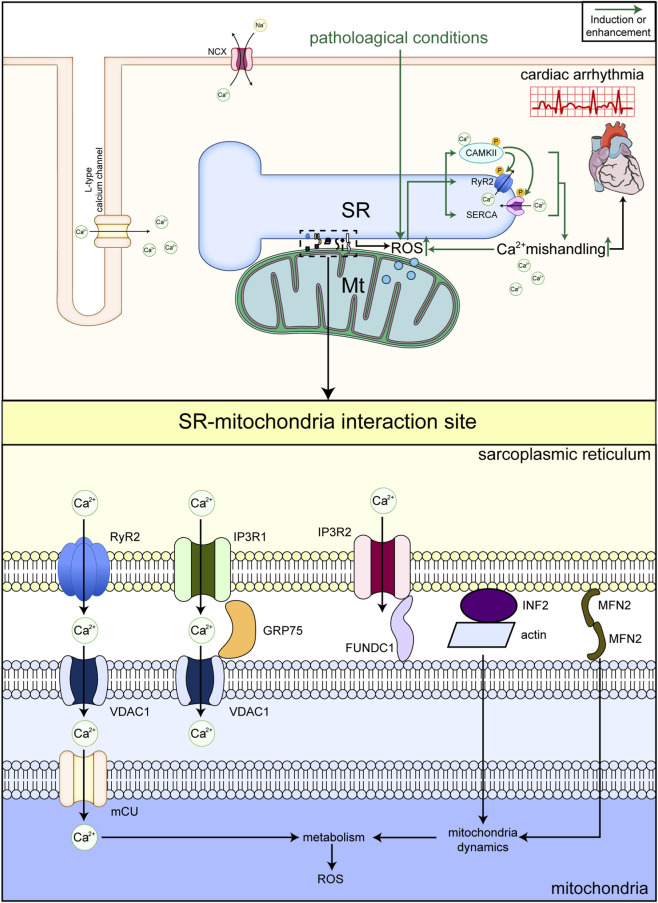
Representative diagram of the interaction between sarcoplasmic reticulum (SR) and mitochondria (Mt) affecting cardiac arrhythmia. In the interaction site between SR and mitochondria (below), Ca^2+^ transfers from SR to mitochondria through two molecular structures: the complex VDAC1/RyR2 and the complex IP3R1/VDAC1/GRP75. The complex IP3R2/FUNDC1 serves as a modulator to mediate Ca^2+^ release. The dimer formed by mitofusin2 (MFN2) and the complex inverted formin 2 (INF2)/actin serve as modulators for mitochondrial dynamics. These molecules jointly regulate metabolism in mitochondria and finally influence mitochondrial ROS production. In pathological conditions, RyR2, SERCA2a and CaMKII are activated after redox modification by ROS and aggravate Ca^2+^ overload. Finally, the positive feedback loop formed by ROS and Ca^2+^ promotes cardiac arrhythmia (above).

Taken together, the SR regulates mitochondrial ROS production, whereas mitochondria influence redox modifications of SR Ca^2+^-handling proteins. SR–mitochondria crosstalk contributes to heart rate control in health and is involved in arrhythmias under oxidative stress (Figure 4).

## 6 Current status and perspectives of cardiac arrhythmia treatments based on the interaction of SR and mitochondria

Cardiac arrhythmias are a group of disorders characterized by abnormal cardiac electrophysiology. Over the past decades, numerous studies have investigated the molecular targets of AF to understand how ectopic impulses originate. However, despite the availability of defibrillators, there remains an urgent need for effective treatments for ventricular fibrillation (VF), given its high lethality, particularly in the setting of arrhythmogenic cardiomyopathy or myocardial ischemia. Pharmacologically, dantrolene significantly prevented CPVT and VF by regulating Ca^2+^-related pathways (Suetomi et al., 2011; Zamiri et al., 2014; Roden and Knollmann, 2014). In addition, meclofenoxate, an anti-arthritic drug, has also shown anti-arrhythmic effects against CPVT by ameliorating Ca^2+^ overload (Vandewiele et al., 2022). Furthermore, empagliflozin is an alternative option to normalize Ca^2+^ handling and attenuate ventricular arrhythmias in diabetic cardiomyopathy (Kadosaka et al., 2023). From the perspective of lifestyle interventions, exercise training appears effective in reducing RyR2 overactivation and protecting against ventricular tachycardia (Manotheepan et al., 2016). Collectively, these studies provide compelling evidence that alleviating Ca^2+^ overload by modulating Ca^2+^-handling proteins in ventricular cardiomyocytes is a promising strategy for VF. In addition to therapies targeting SR calcium signaling, mitochondrial-targeted drugs offer a novel perspective for treating arrhythmias. MitoQ can be reduced to ubiquinol by mitochondrial respiratory chain complexes I and II, thereby inhibiting the production of mitochondrial reactive oxygen species (mtROS) and creating conditions for alleviating myocardial structural remodeling and preventing arrhythmias in experimental settings. Meanwhile, SS-31 binds to cardiolipin on the mitochondrial inner membrane, protecting it from oxidative damage in preclinical studies (Goodman et al., 2020). It is noted that the clinical application of these drugs specifically targeting SR-mitochondria crosstalk remains to be investigated. Additionnally, metabolic modulators like ranolazine are also reported to promote the shift from fatty acid oxidation to glucose oxidation in cardiomyocytes and reducing oxidative stress (Kourampi et al., 2023). These agents demonstrate that mitochondrial-targeted drugs also possess anti-arrhythmic potential.

Currently, therapeutic strategies targeting SR–mitochondria interactions primarily rely on indirect approaches, aiming to ameliorate related pathological conditions by restoring calcium homeostasis and alleviating oxidative stress. However, the development of specific agents capable of directly targeting ER–mitochondria contact sites to more precisely restore their structural integrity and functional balance remains an unmet need.

This paper reviews the significance of SR–mitochondria interactions in arrhythmia development and suggests that these contacts are key(Bariani et al., 2024; Zeppenfeld et al., 2022) determinants of Ca^2+^ homeostasis. However, there are still few treatments that specifically target core components at SR–mitochondria contact sites. Xestospongin B, an IP_3_R inhibitor, has been shown to modulate Ca^2+^ transfer mediated by MAMs, thereby conferring cardioprotection (Zhang et al., 2023). Metformin indirectly modulates the expression of MAMs-associated proteins such as GRP75 and MFN2 by activating AMPK, thereby alleviating ERS and improving cardiomyocyte function (Angebault et al., 2021). Encouragingly, advances in protein structural analysis may enable more precise therapeutic design and are expected to accelerate the development of targeted drugs. Given the importance of SR–mitochondria interactions in arrhythmia onset, the molecular components at SR–mitochondria contact sites warrant greater attention. For example, targeting RyR2 (Min et al., 2012) and Mfn1/2 (Zhang et al., 2024) presents promising therapeutic strategies for cardiovascular diseases through the restoration of normal SR-mito crosstalk. Therapeutics targeting these molecules may further modulate mitochondrial metabolism, mitochondrial dynamics, and mitophagy, which are critical for cardiomyocyte survival and function. Likewise, SR Ca^2+^ storage, release, and uptake may be rebalanced by such targeted interventions in patients with cardiac arrhythmias. Although substantial work remains before clinical translation, we believe that therapies based on mechanisms associated with SR–mitochondria interactions are promising. In conclusion, we propose that SR–mitochondria crosstalk represents a novel mechanistic axis in cardiac arrhythmias and provides potential molecular targets for therapy.

## 7 Conclusion

The interaction between the SR and mitochondria represents a promising avenue for exploring the onset and progression of cardiac arrhythmias; thus, basic and clinical research targeting this interaction will be instrumental in advancing therapeutic strategies.
